# Association of the C-reactive protein-triglyceride glucose index with microvascular obstruction and long-term prognosis in patients with acute myocardial infarction: a CMR-based study

**DOI:** 10.3389/fendo.2026.1889147

**Published:** 2026-07-15

**Authors:** Lei Chen, Dexiang Zong, Peng Lu, Temilola J. Oketunbi, Mingchang Du, Donglin Liu, Xueyuan Qi, Wenliang Che, Yuan Lu, Wensu Chen

**Affiliations:** 1 Department of Cardiology, The Affiliated Hospital of Xuzhou Medical University, Xuzhou, China; 2 National Heart Centre Singapore, Duke - NUS Medical School, Singapore, Singapore; 3 Department of Cardiology, Shanghai Tenth People’s Hospital, Tongji University School of Medicine, Shanghai, China

**Keywords:** acute myocardial infarction, cardiac magnetic resonance, insulin resistance, microvascular obstruction, systemic inflammation

## Abstract

**Background:**

The clinical benefits of percutaneous coronary intervention (PCI) for acute myocardial infarction (AMI) are frequently compromised by the occurrence of microvascular obstruction (MVO). The C-reactive protein-triglyceride glucose index (CTI) is a novel biomarker integrating systemic inflammation and insulin resistance. We aimed to evaluate the association between CTI and MVO, as well as its prognostic value for long-term outcomes in AMI patients.

**Methods:**

This study included patients with AMI who underwent successful primary PCI and subsequent cardiac magnetic resonance (CMR). MVO and cardiac function were assessed by CMR. The primary endpoint was the occurrence of MVO, and the secondary endpoint was the incidence of major adverse cardiovascular events (MACE).

**Results:**

A total of 967 patients were ultimately included, comprising 255 with diabetes mellitus and 712 without diabetes; 476 patients developed CMR-defined MVO. Higher baseline CTI levels were significantly and independently associated with an increased risk of MVO (OR = 1.33, 95% CI: 1.13–1.56, P = 0.001). A linear dose-response relationship was observed between CTI and MVO risk (P for overall < 0.05, P for nonlinear > 0.05). During a median follow-up of 43 months, higher CTI levels were associated with an increased risk of long-term MACE in the overall population and the non-diabetic subgroup, whereas this association was attenuated in patients with diabetes mellitus.

**Conclusions:**

CTI is independently associated with MVO after PCI in AMI patients and shows modest prognostic value for long-term MACE, particularly in patients without diabetes. As a clinically accessible tool, CTI may facilitate early risk stratification and individualized microvascular protection strategies following PCI.

## Introduction

Microvascular obstruction (MVO) is a frequent and clinically important complication after percutaneous coronary intervention (PCI) for acute myocardial infarction (AMI), and is closely associated with infarct expansion, adverse left ventricular remodeling, heart failure, and poor long-term outcomes (1–4). Given its important clinical implications, the latest expert consensus from the Canadian Cardiovascular Society (CCS) has explicitly endorsed the use of MVO for risk stratification in AMI, further establishing its pivotal role in clinical decision-making (5). However, although cardiac magnetic resonance (CMR) is the gold standard for diagnosing MVO, its widespread application in critically ill patients during the acute phase of AMI is limited by logistical complexity and equipment accessibility (6). Therefore, identifying simple and clinically accessible biomarkers associated with MVO may help improve early risk stratification after AMI.

The pathological mechanisms underlying MVO are multifaceted, involving reperfusion injury, microvascular spasm, endothelial dysfunction, and inflammatory responses (7). Insulin resistance and systemic inflammation are particularly important contributors to coronary microvascular dysfunction (8, 9). The triglyceride-glucose (TyG) index is a convenient surrogate marker of insulin resistance and has been associated with coronary microvascular dysfunction and adverse cardiovascular outcomes (10). C-reactive protein (CRP), as a marker of systemic inflammation, is also widely used in cardiovascular risk assessment. However, TyG and CRP each capture only one dimension of the complex metabolic-inflammatory process involved in microvascular injury. The C-reactive protein-triglyceride glucose index (CTI) integrates inflammatory burden and insulin resistance into a single composite indicator. Previous studies have suggested that CTI is associated with adverse outcomes in stroke, coronary artery disease, and cardiometabolic multimorbidity (11–14). Nevertheless, the intrinsic association between CTI and the risk of MVO following PCI in AMI patients has not been systematically elucidated.

Therefore, this study aimed to evaluate the association between baseline CTI levels and CMR-defined MVO in patients with AMI after PCI. We further examined whether this association differed according to diabetes status and assessed the prognostic value of CTI for long-term major adverse cardiovascular events (MACE).

## Methods

### Study design and population

**Figure 1:**
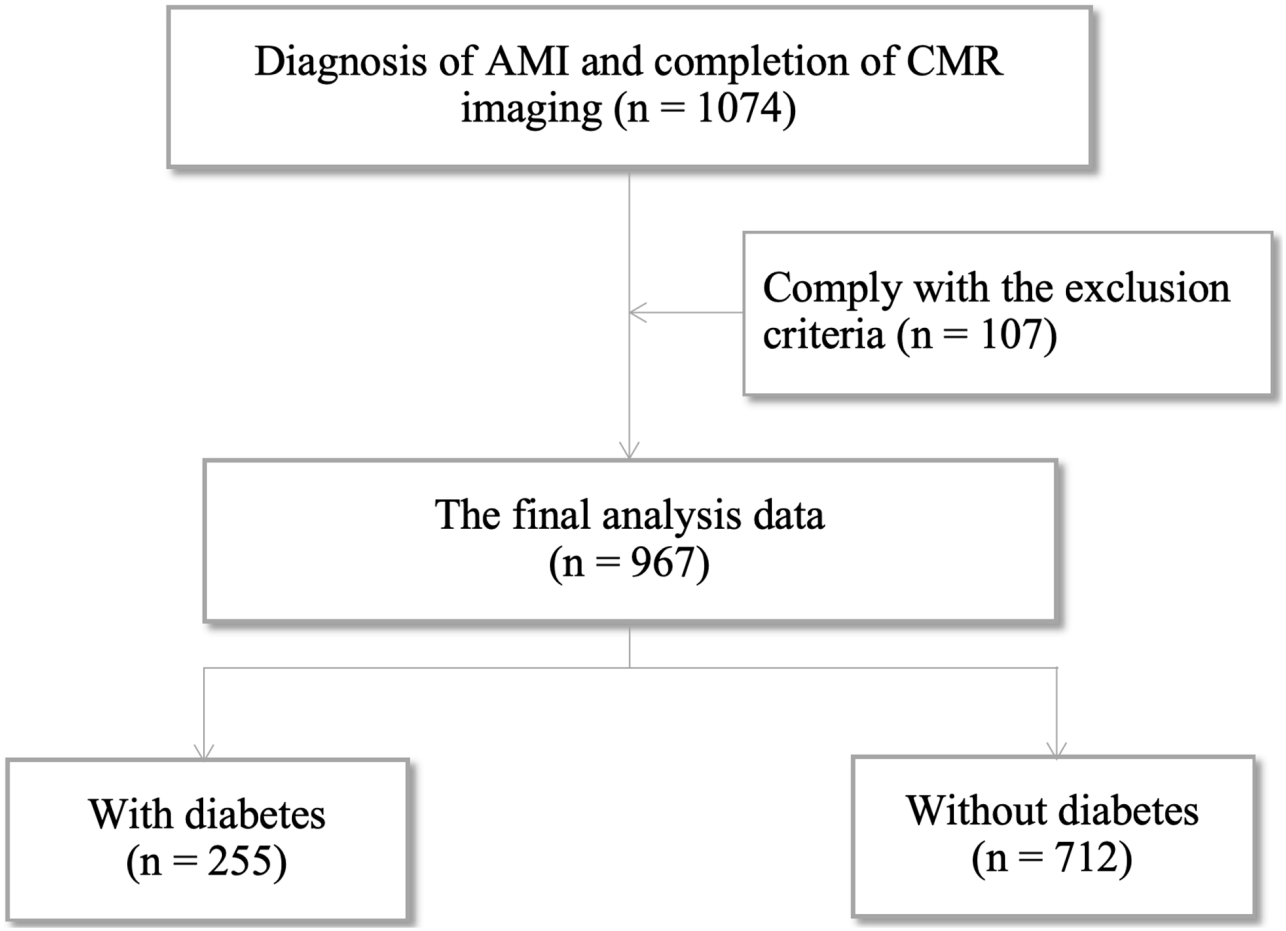
Study flowchart. AMI, acute myocardial infarction; CMR, cardiac magnetic resonance.

This was a single-center retrospective observational study that consecutively enrolled AMI patients admitted to the Affiliated Hospital of Xuzhou Medical University between January 2019 and October 2025. AMI was diagnosed according to the Fourth Universal Definition of Myocardial Infarction (15). The inclusion criteria were as follows: (1) successful primary PCI (pPCI) performed within 12 hours of symptom onset for ST-segment elevation myocardial infarction (STEMI) patients, or according to an early invasive strategy based on risk stratification for non-ST-segment elevation myocardial infarction (NSTEMI) patients, with a post-procedural Thrombolysis in Myocardial Infarction (TIMI) flow grade of 3 in the infarct-related artery (IRA); (2) completion of CMR imaging during hospitalization; and (3) availability of complete clinical data. The exclusion criteria were: (1) poor CMR image quality precluding accurate post-processing analysis; (2) a history of prior myocardial infarction; (3) concomitant malignancy or systemic active inflammatory disease; (4) severe hepatic or renal insufficiency; (5) type 1 diabetes mellitus; and (6) corticosteroid use or thyroid dysfunction. The study protocol was approved by the Ethics Review Committee of the Affiliated Hospital of Xuzhou Medical University (No. XYFY2026 - KL154 - 01). Given the retrospective design and the absence of additional risk to patients, the requirement for written informed consent was waived by the ethics committee. A total of 967 patients were ultimately included (**Figure 1**).

### Data collection and variable definitions

Baseline demographic characteristics, medical history (hypertension, diabetes, stroke, etc.), in-hospital medication information (including statins, antiplatelet agents, angiotensin-converting enzyme inhibitors/angiotensin receptor blockers [ACEI/ARB], beta-blockers, and sodium-glucose cotransporter 2 [SGLT2] inhibitors), laboratory parameters (including fasting blood glucose [FBG], total cholesterol [TC], triglycerides [TG], high-sensitivity C-reactive protein [hs-CRP], high-sensitivity troponin T [hsTnT], N-terminal pro-B-type natriuretic peptide [NT-proBNP], etc.), and coronary angiographic characteristics (pre-procedural TIMI flow grade, IRA) were collected from the hospital electronic medical record system. Diabetes mellitus was defined according to documented medical history, use of glucose-lowering therapy, or laboratory findings meeting established diagnostic criteria for diabetes (16). CTI was calculated using the following formula (11–14): CTI = 0.412 × Ln (hs-CRP [mg/L]) + Ln (TG [mg/dL] × FBG [mg/dL])/2. Because TG and FBG are commonly reported in mmol/L, including in our laboratory, values were converted to mg/dL before CTI calculation. Based on admission CTI levels, the total population was divided into tertiles: a low-CTI group (n = 322), a middle-CTI group (n = 303), and a high-CTI group (n = 342). Given the skewed distribution, hsTnT and NT-proBNP were log-transformed before inclusion in regression analyses.

### CMR protocol and image analysis

All patients underwent CMR examination at a median of 4 days (interquartile range: 3–6 days) after admission. Imaging was performed on a 3.0 T superconducting magnetic resonance scanner (Ingenia, Philips, The Netherlands) using a balanced steady-state free precession (bSSFP) sequence for cine image acquisition. The detailed scanning protocol has been described in our previously published work (17). Post-processing analysis of CMR images was uniformly performed using CVI42 software (version 5.13.5, Circle Cardiovascular Imaging, Canada). All images were independently analyzed by two physicians — a radiologist and a cardiovascular specialist, each with 10 years of experience — who were blinded to all clinical data. The software automatically delineated and calculated left ventricular end-diastolic volume (EDV), end-systolic volume (ESV), and left ventricular ejection fraction (LVEF), all indexed to body surface area (BSA). Global longitudinal strain (GLS) was derived using CMR feature tracking technology. MVO and infarct areas were identified on late gadolinium enhancement (LGE) images. LGE was defined as regions with signal intensity exceeding 5 standard deviations (5 SD) above the mean signal of remote normal myocardium. MVO was defined as a hypointense core within the hyperenhanced LGE region (18). Both LGE and MVO sizes were expressed as a percentage of total left ventricular myocardial mass.

### Clinical follow-up and endpoint definitions

Patients were followed up from discharge through outpatient visits and/or telephone interviews (using a standardized questionnaire). When patients could not be contacted directly, relevant information was obtained from their family members or attending physicians. The primary endpoint was CMR-confirmed MVO. The secondary endpoint was MACE, defined as a composite of all-cause death, recurrent non-fatal myocardial infarction, and hospitalization for heart failure. All-cause death was defined as death from any cause during follow-up. Recurrent myocardial infarction was defined according to the Fourth Universal Definition of Myocardial Infarction as a new ischemic event with elevated cardiac troponin and supporting clinical, electrocardiographic, imaging, or angiographic evidence. Hospitalization for heart failure was defined as an unplanned admission for worsening heart failure symptoms or signs requiring intensified treatment. Stroke was defined as neurological dysfunction and cerebrovascular injury caused by cerebral ischemia or cerebral hemorrhage. These definitions were based on ESC guidelines and standardized cardiovascular endpoint definitions (15, 19).

### Statistical analysis

Statistical analysis was performed using SPSS 26.0 (IBM, Chicago, USA) and R software (Lucent Technologies, New Jersey, USA). The normality of continuous variables was assessed using the Kolmogorov-Smirnov test. Normally distributed continuous variables were expressed as mean ± standard deviation and compared using independent-samples t-test or one-way analysis of variance (ANOVA); non-normally distributed variables were expressed as median and interquartile range and compared using the Mann-Whitney U test or Kruskal-Wallis test. Categorical variables were expressed as frequencies and percentages and compared using the chi-square test. Variables with P < 0.05 in univariable regression analysis along with established traditional risk factors reported in the literature were entered into a multivariable logistic regression model using forward stepwise selection to evaluate the independent association between CTI levels and MVO occurrence. The entry and removal criteria were set at P < 0.05 and P > 0.10, respectively. Variables that did not meet the retention criterion were automatically excluded from the final equation. Therefore, only variables retained in the final stepwise model are presented in the results. Odds ratios (OR) with 95% confidence intervals (CI) were calculated in the overall population, diabetic subgroup, and non-diabetic subgroup, respectively. Different adjustment models were constructed in the multivariable analysis: Model 1 was primarily adjusted for clinical and laboratory parameters; Model 2 was further adjusted for the imaging parameters LGE, GLS, and LVEF on the basis of Model 1. Restricted cubic spline (RCS) analysis was used to examine the dose-response relationship between CTI and MVO risk. Bootstrap internal validation with 200 resamples was performed to assess the optimism-corrected AUC, calibration intercept, and calibration slope of the MVO model. Kaplan-Meier survival curves were plotted, and the log-rank test was used to compare long-term MACE-free survival rates across different CTI groups. Cox proportional hazards regression was used to evaluate the association between CTI as a continuous variable and long-term MACE, adjusting for age, sex, STEMI, diabetes mellitus, systolic blood pressure, heart rate, NT-proBNP, and hsTnT. Diabetes mellitus was not adjusted for in subgroup analyses stratified by diabetes status. Time-dependent ROC analysis was used to assess the predictive performance of CTI for MACE at 1, 2, and 3 years. All statistical tests were two-sided, and a P value < 0.05 was considered statistically significant.

## Results

### Baseline clinical characteristics

A total of 967 AMI patients who underwent PCI were enrolled, with a mean age of 58.00 ± 11.74 years, including 795 males (82.21%), comprising 255 with type 2 diabetes mellitus and 712 without diabetes. Of these, 643 were diagnosed with STEMI and 324 with NSTEMI. Compared with the non-diabetic group, the diabetic group had a significantly higher proportion of stroke (14.51% vs. 9.27%, P = 0.020), faster heart rate (81.30 ± 14.79 vs. 77.64 ± 12.35 bpm, P < 0.001), higher hs-CRP levels [21.90 (7.75, 70.25) vs. 13.65 (5.00, 34.32) mg/L, P < 0.001], greater LGE% (48.10 ± 29.81 vs. 40.23 ± 26.47, P < 0.001), and lower absolute GLS values (11.25 ± 4.29% vs. 12.23 ± 4.24%, P = 0.002). CTI was significantly higher in the diabetic group than in the non-diabetic group (10.55 ± 0.98 vs. 9.83 ± 0.82, P < 0.001), as were the TyG index (9.30 ± 0.74 vs. 8.77 ± 0.59, P < 0.001) and FBG (8.73 ± 3.18 vs. 5.86 ± 1.72 mmol/L, P < 0.001). The incidence of MVO was significantly higher in the diabetic group (58.04% vs. 46.07%, P = 0.001), and MVO% was also greater [3.52 (0.00, 7.55) vs. 0.00 (0.00, 6.06), P < 0.001]. The utilization rate of SGLT2 inhibitors was significantly higher in the diabetic group (63.53% vs. 13.20%, P < 0.001) (**Table 1**).

**Table 1:** Baseline patient characteristics.

Variables	Total (n = 967)	Non-diabetes (n = 712)	Diabetes (n = 255)	*P*
Male, n (%)	795 (82.21)	591 (83.01)	204 (80.00)	0.281
Age (years)	58.00 ± 11.74	57.67 ± 12.06	58.94 ± 10.78	0.117
BMI (kg/m^2^)	25.78 ± 3.81	25.74 ± 3.96	25.89 ± 3.35	0.585
Smoking,n (%)	495 (51.19)	366 (51.40)	129 (50.59)	0.823
Hypertension,n (%)	461 (47.67)	331 (46.49)	130 (50.98)	0.218
Stroke,n (%)	103 (10.65)	66 (9.27)	37 (14.51)	0.020
Heart rate, bpm	78.61 ± 13.13	77.64 ± 12.35	81.30 ± 14.79	<.001
SBP (mmHg)	127.82 ± 18.92	127.63 ± 18.93	128.33 ± 18.94	0.611
DBP (mmHg)	80.48 ± 13.06	80.94 ± 13.14	79.21 ± 12.76	0.070
LGE%	42.30 ± 27.59	40.23 ± 26.47	48.10 ± 29.81	<.001
LVESVI, mL/m^2^	44.07 ± 17.35	43.67 ± 17.45	45.19 ± 17.04	0.231
LVEDVI, mL/m^2^	78.88 ± 20.99	78.27 ± 21.19	80.60 ± 20.39	0.128
LVEF, %	52.15 ± 8.44	52.16 ± 8.47	52.10 ± 8.36	0.916
LV mass, g	111.69 ± 27.67	111.76 ± 27.54	111.48 ± 28.08	0.887
GLS, %	11.97 ± 4.27	12.23 ± 4.24	11.25 ± 4.29	0.002
MVO%	0.00 (0.00, 6.61)	0.00 (0.00, 6.06)	3.52 (0.00, 7.55)	<.001
MVO, n (%)	476 (49.22)	328 (46.07)	148 (58.04)	0.001
NT-proBNP (pg/ml)	1046.00 (518.00, 1932.50)	1057.00 (511.50, 1936.68)	1040.53 (545.50, 1918.22)	0.670
hsTnT (ng/ml)	2535.00 (888.50, 5337.50)	2467.00 (821.00, 5170.75)	2807.00 (1044.00, 5811.50)	0.166
hs-CRP, mg/L	15.10 (5.60, 43.65)	13.65 (5.00, 34.32)	21.90 (7.75, 70.25)	<.001
eGFR, mL/min/1.73m^2^	108.02 ± 16.05	107.62 ± 15.40	109.16 ± 17.73	0.189
CTI	10.02 ± 0.92	9.83 ± 0.82	10.55 ± 0.98	<.001
TyG	8.91 ± 0.67	8.77 ± 0.59	9.30 ± 0.74	<.001
FBG, mmol/L	6.62 ± 2.54	5.86 ± 1.72	8.73 ± 3.18	<.001
TC (mmol/L)	4.33 ± 1.07	4.31 ± 1.09	4.40 ± 1.02	0.271
TG (mmol/L)	1.77 ± 1.36	1.67 ± 1.06	2.06 ± 1.92	0.002
LDL-C (mmol/L)	2.92 ± 6.44	3.00 ± 7.49	2.68 ± 0.92	0.493
HDL-C (mmol/L)	0.96 ± 0.25	0.97 ± 0.25	0.95 ± 0.25	0.268
Preprocedural TIMI flow gradeda ≤ 1, n (%)	741 (76.63)	540 (75.84)	201 (78.82)	0.334
STEMI, n (%)	643 (66.49)	465 (65.31)	178 (69.80)	0.192
MACE, n (%)	256 (26.47)	180 (25.28)	76 (29.80)	0.160
IRA-LCX, n (%)	181 (18.72)	136 (19.10)	45 (17.65)	0.609
IRA-LAD, n (%)	481 (49.74)	355 (49.86)	126 (49.41)	0.902
IRA-RCA, n (%)	298 (30.82)	217 (30.48)	81 (31.76)	0.702
IRA-LM, n (%)	4 (0.41)	2 (0.28)	2 (0.78)	0.613
IRA-RM, n (%)	3 (0.31)	2 (0.28)	1 (0.39)	1.000
SGLT2 inhibitor, n (%)	256 (26.47)	94 (13.20)	162 (63.53)	<.001
β-blockers, n (%)	829 (85.73)	601 (84.41)	228 (89.41)	0.050
ACEI/ARB, n (%)	614 (63.50)	448 (62.92)	166 (65.10)	0.536
Aspirin, n (%)	892 (92.24)	655 (91.99)	237 (92.94)	0.628
P2Y12, n (%)	916 (94.73)	671 (94.24)	245 (96.08)	0.260

BMI, body mass index; GFR, glomerular filtration rate; LVEF, left ventricular ejection fraction; GLS, global longitudinal strain; LV, left ventricular; EDVi, end-diastolic volume index; LGE, late gadolinium enhanced; ESVi, end-systolic volume index; SBP, systolic blood pressure; DBP, diastolic blood pressure; IRA, infarct-related artery; LAD, left anterior descending artery; LCX, left circumﬂex artery; RCA, right coronary artery; LM, left main coronary artery; RM, right main coronary artery; HDL-C, high-density lipoprotein cholesterol; LDL-C, low-density lipoprotein cholesterol; hs-CRP, high sensitivity C-reactive protein; hs-TnT, high sensitivity troponin T; NT-proBNP, N-terminal pro-B-type natriuretic peptide; ACEI, angiotensin-converting-enzyme inhibitor; ARB, angiotensin II receptor; SGLT2, sodium-dependent glucose transporters 2; TC, total cholesterol; TG, triglycerides; STEMI, ST-segment elevation myocardial infarction; TyG, triglyceride-glucose index; TIMI, Thrombolysis in Myocardial Infarction flow grade; MACE, major adverse cardiovascular events; FBG, fasting blood glucose; CTI, C-reactive protein–triglyceride glucose index.

### Baseline characteristics by CTI tertiles

Patients were divided into a low-CTI group (n = 322, CTI 9.02 ± 0.45), a middle-CTI group (n = 303, CTI 9.99 ± 0.22), and a high-CTI group (n = 342, CTI 10.99 ± 0.54) based on CTI tertiles. With increasing CTI levels, patients were older (55.86 vs. 56.83 vs. 61.07 years, P < 0.001), had higher body mass index (BMI) (25.22 vs. 25.46 vs. 26.58 kg/m², P < 0.001), a progressively higher prevalence of diabetes (11.49% vs. 20.79% vs. 45.32%, P < 0.001), and faster heart rates (P = 0.003). Regarding CMR parameters, LGE% was significantly higher in the high-CTI group (48.58 ± 27.69%) than in the low-CTI group (34.47 ± 24.81%), LVEF decreased progressively across groups (53.28% vs. 52.12% vs. 51.09%, P = 0.004), and absolute GLS values decreased across groups (13.17% vs. 12.30% vs. 10.55%, P < 0.001). The incidence of MVO exhibited a significant increasing trend with higher CTI (37.58% vs. 49.17% vs. 60.23%, P < 0.001), and MVO% followed a similar pattern [0.00 (0.00, 4.75) vs. 0.00 (0.00, 6.82) vs. 3.61 (0.00, 7.45), P < 0.001]. Laboratory parameters including hs-CRP, hsTnT, NT-proBNP, FBG, and TG all increased significantly with higher CTI. The incidence of MACE in the low-, middle-, and high-CTI groups was 19.88%, 26.07%, and 33.04%, respectively (P < 0.001). The proportion of pre-procedural TIMI flow ≤ 1 also increased with higher CTI (70.81% vs. 76.57% vs. 82.16%, P = 0.003) (**Table 2**).

**Table 2:** Baseline patient characteristics based on CTI.

Variables	Low CTI (n = 322)	Medium CTI (n = 303)	High CTI (n = 342)	*P*
Male, n (%)	264 (81.99)	256 (84.49)	275 (80.41)	0.398
Age (years)	55.86 ± 11.85	56.83 ± 12.31	61.07 ± 10.44	<.001
BMI (kg/m^2^)	25.22 ± 4.13	25.46 ± 2.98	26.58 ± 4.01	<.001
Smoking, n (%)	154 (47.83)	164 (54.13)	177 (51.75)	0.280
Hypertension, n (%)	154 (47.83)	132 (43.56)	175 (51.17)	0.155
Diabetes, n (%)	37 (11.49)	63 (20.79)	155 (45.32)	<.001
Stroke, n (%)	38 (11.80)	29 (9.57)	36 (10.53)	0.662
Heart rate, bpm	76.72 ± 12.24	78.90 ± 12.06	80.12 ± 14.59	0.003
SBP (mmHg)	129.21 ± 18.82	126.99 ± 18.15	127.24 ± 19.66	0.267
DBP (mmHg)	80.71 ± 12.23	80.23 ± 12.72	80.49 ± 14.11	0.900
LGE%	34.47 ± 24.81	43.53 ± 28.35	48.58 ± 27.69	<.001
LVESVI, mL/m^2^	43.35 ± 18.18	43.45 ± 16.96	45.31 ± 16.87	0.261
LVEDVI, mL/m^2^	77.52 ± 19.62	77.42 ± 20.02	81.46 ± 22.82	0.018
LVEF, %	53.28 ± 7.77	52.12 ± 9.15	51.09 ± 8.27	0.004
LV mass, g	112.88 ± 26.38	112.09 ± 25.96	110.21 ± 30.21	0.441
GLS, %	13.17 ± 4.08	12.30 ± 4.02	10.55 ± 4.27	<.001
MVO%	0.00 (0.00,4.75)	0.00 (0.00,6.82)	3.61 (0.00,7.45)	<.001
MVO, n (%)	121 (37.58)	149 (49.17)	206 (60.23)	<.001
NT-proBNP (pg/ml)	955.64 (444.88,1730.75)	979.00 (510.00,1932.50)	1175.50 (608.41,2255.27)	<.001
hsTnT (ng/ml)	1859.00 (607.00,4651.25)	2345.00 (819.00,4453.00)	3475.00 (1277.25,6827.50)	<.001
hs-CRP, mg/L	4.45 (2.00,10.57)	14.90 (7.70,30.70)	48.70 (21.70,90.12)	<.001
eGFR, mL/min/1.73m^2^	108.44 ± 14.91	108.56 ± 15.47	107.15 ± 17.54	0.458
CTI	9.02 ± 0.45	9.99 ± 0.22	10.99 ± 0.54	<.001
TyG	8.36 ± 0.45	8.87 ± 0.41	9.47 ± 0.58	<.001
FBG, mmol/L	5.51 ± 1.37	6.40 ± 2.03	7.85 ± 3.19	<.001
TC (mmol/L)	4.14 ± 1.07	4.23 ± 0.99	4.61 ± 1.09	<.001
TG (mmol/L)	1.10 ± 0.49	1.58 ± 0.67	2.58 ± 1.86	<.001
LDL-C (mmol/L)	3.22 ± 11.09	2.68 ± 0.88	2.84 ± 0.91	0.555
HDL-C (mmol/L)	1.03 ± 0.26	0.96 ± 0.26	0.89 ± 0.22	<.001
Preprocedural TIMI flow gradeda ≤ 1, n (%)	228 (70.81)	232 (76.57)	281 (82.16)	0.003
STEMI, n (%)	215 (66.77)	186 (61.39)	242 (70.76)	0.042
MACE, n (%)	64 (19.88)	79 (26.07)	113 (33.04)	<.001
IRA-LCX, n (%)	48 (14.91)	60 (19.80)	73 (21.35)	0.088
IRA-LAD, n (%)	177 (54.97)	143 (47.19)	161 (47.08)	0.071
IRA-RCA, n (%)	95 (29.50)	99 (32.67)	104 (30.41)	0.678
IRA-LM, n (%)	1 (0.31)	1 (0.33)	2 (0.58)	1.000
IRA-RM, n (%)	1 (0.31)	0 (0.00)	2 (0.58)	0.778
Killip class>1, n (%)	29 (9.01)	20 (6.60)	34 (9.94)	0.302
Statins, n (%)	297 (92.24)	292 (96.37)	326 (95.32)	0.056
SGLT2 inhibitor, n (%)	47 (14.60)	60 (19.80)	149 (43.57)	<.001
β-blockers, n (%)	266 (82.61)	263 (86.80)	300 (87.72)	0.139
ACEI/ARB, n (%)	192 (59.63)	203 (67.00)	219 (64.04)	0.155
Aspirin, n (%)	291 (90.37)	286 (94.39)	315 (92.11)	0.171
P2Y12, n (%)	302 (93.79)	287 (94.72)	327 (95.61)	0.575

BMI, body mass index; GFR, glomerular filtration rate; LVEF, left ventricular ejection fraction; GLS, global longitudinal strain; LV, left ventricular; EDVi, end-diastolic volume index; LGE, late gadolinium enhanced; ESVi, end-systolic volume index; SBP, systolic blood pressure; DBP, diastolic blood pressure; IRA, infarct-related artery; LAD, left anterior descending artery; LCX, left circumﬂex artery; RCA, right coronary artery; LM, left main coronary artery; RM, right main coronary artery; HDL-C, high-density lipoprotein cholesterol; LDL-C, low-density lipoprotein cholesterol; hs-CRP, high sensitivity C-reactive protein; hs-TnT, high sensitivity troponin T; NT-proBNP, N-terminal pro-B-type natriuretic peptide; ACEI, angiotensin-converting-enzyme inhibitor; ARB, angiotensin II receptor; SGLT2, sodium-dependent glucose transporters 2; TC, total cholesterol; TG, triglycerides; STEMI, ST-segment elevation myocardial infarction; TyG, triglyceride-glucose index; TIMI, Thrombolysis in Myocardial Infarction flow grade; MACE, major adverse cardiovascular events; FBG, fasting blood glucose; CTI, C-reactive protein–triglyceride glucose index.

### Logistic regression analysis for MVO in the overall population

Univariable logistic regression analysis (**Table 3**) showed that each one-unit increase in CTI was associated with a 65% increase in MVO risk (OR = 1.65, 95% CI: 1.37–2.00, P < 0.001). In the tertile-based analysis, compared with the low-CTI group, the middle-CTI group had a 73% higher MVO risk (OR = 1.73, 95% CI: 1.22–2.46, P = 0.002) and the high-CTI group had a 160% higher MVO risk (OR = 2.60, 95% CI: 1.78–3.80, P < 0.001). Other factors significantly associated with MVO included diabetes, heart rate, systolic blood pressure (SBP), LGE%, GLS, hsTnT, hs-CRP, TyG, FBG, TG, MACE, pre-procedural TIMI ≤ 1, STEMI, IRA–left circumflex artery (LCX), and IRA–left anterior descending artery (LAD).

**Table 3:** Univariate logistic regression analysis for MVO in all patients.

Variables	OR (95%CI)	*P*
Male, n (%)	0.97 (0.66 ~ 1.44)	0.882
Age (years)	1.00 (0.99 ~ 1.01)	0.765
BMI (kg/m^2^)	0.99 (0.95 ~ 1.03)	0.593
Smoking, n (%)	1.27 (0.94 ~ 1.70)	0.118
Hypertension, n (%)	0.94 (0.73 ~ 1.21)	0.613
Diabetes, n (%)	1.62 (1.21 ~ 2.16)	0.001
Stroke, n (%)	0.97 (0.59 ~ 1.62)	0.916
Heart rate, bpm	1.02 (1.01 ~ 1.03)	0.008
SBP (mmHg)	0.99 (0.98 ~ 0.99)	0.011
DBP (mmHg)	1.00 (0.99 ~ 1.01)	0.646
LGE%	1.02 (1.01 ~ 1.02)	<.001
LVESVI, mL/m^2^	1.00 (0.99 ~ 1.01)	0.599
LVEDVI, mL/m^2^	1.00 (1.00 ~ 1.01)	0.550
LVEF, %	0.99 (0.97 ~ 1.00)	0.129
LV mass, g	1.00 (0.99 ~ 1.00)	0.384
GLS, %	0.94 (0.91 ~ 0.97)	<.001
NT-proBNP (pg/ml)	1.13 (0.99 ~ 1.30)	0.072
hsTnT (ng/ml)	1.63 (1.44 ~ 1.85)	<.001
hs-CRP, mg/L	1.01 (1.01 ~ 1.01)	<.001
eGFR, mL/min/1.73m^2^	1.00 (0.99 ~ 1.01)	0.541
CTI	1.65 (1.37 ~ 2.00)	<.001
TyG	1.32 (1.03 ~ 1.70)	0.029
FBG, mmol/L	1.14 (1.04 ~ 1.24)	0.006
TC (mmol/L)	1.06 (0.93 ~ 1.22)	0.384
TG (mmol/L)	1.18 (1.02 ~ 1.36)	0.027
LDL-C (mmol/L)	1.07 (0.91 ~ 1.26)	0.401
HDL-C (mmol/L)	1.50 (0.83 ~ 2.70)	0.176
Preprocedural TIMI ≤1, n (%)	1.84 (1.29 ~ 2.63)	<.001
CTI groups, n (%)		
Low CTI	1.00 (Reference)	
Medium CTI	1.73 (1.22 ~ 2.46)	0.002
High CTI	2.60 (1.78 ~ 3.80)	<.001
STEMI, n (%)	2.20 (1.60 ~ 3.03)	<.001
MACE, n (%)	2.13 (1.51 ~ 3.00)	<.001
IRA-LCX, n (%)	0.65 (0.44 ~ 0.95)	0.026
IRA-LAD, n (%)	0.65 (0.44 ~ 0.95)	0.026
IRA-RCA, n (%)	0.79 (0.57 ~ 1.09)	0.143
IRA-LM, n (%)	0.00 (0.00 ~ Inf)	0.972
IRA-RM, n (%)	0.00 (0.00 ~ Inf)	0.972
Killip class>1, n (%)	1.59 (0.93 ~ 2.71)	0.087
Statins, n (%)	0.85 (0.45 ~ 1.58)	0.598
SGLT2 inhibitor, n (%)	2.30 (1.47 ~ 3.61)	<.001
β-blockers, n (%)	1.25 (0.83 ~ 1.88)	0.288
ACEI/ARB, n (%)	0.84 (0.62 ~ 1.13)	0.251
Aspirin, n (%)	0.94 (0.55 ~ 1.62)	0.837
P2Y12, n (%)	0.89 (0.47 ~ 1.67)	0.720

BMI, body mass index; GFR, glomerular filtration rate; LVEF, left ventricular ejection fraction; GLS, global longitudinal strain; LV, left ventricular; EDVi, end-diastolic volume index; LGE, late gadolinium enhanced; ESVi, end-systolic volume index; SBP, systolic blood pressure; DBP, diastolic blood pressure; IRA, infarct-related artery; LAD, left anterior descending artery; LCX, left circumﬂex artery; RCA, right coronary artery; LM, left main coronary artery; RM, right main coronary artery; HDL-C, high-density lipoprotein cholesterol; LDL-C, low-density lipoprotein cholesterol; hs-CRP, high sensitivity C-reactive protein; hs-TnT, high sensitivity troponin T; NT-proBNP, N-terminal pro-B-type natriuretic peptide; ACEI, angiotensin-converting-enzyme inhibitor; ARB, angiotensin II receptor; SGLT2, sodium-dependent glucose transporters 2; TC, total cholesterol; TG, triglycerides; STEMI, ST-segment elevation myocardial infarction; TyG, triglyceride-glucose index; TIMI, Thrombolysis in Myocardial Infarction flow grade; MACE, major adverse cardiovascular events; FBG, fasting blood glucose; CTI, C-reactive protein–triglyceride glucose index.

In the multivariable logistic regression analysis (**Table 4**), Model 1, adjusted for age, STEMI, IRA-LAD, pre-procedural TIMI flow, diabetes, SBP, heart rate, NT-proBNP, and hsTnT, showed that CTI remained an independent risk factor for MVO (OR = 1.48, 95% CI: 1.27–1.73, P < 0.001). In Model 2, which further adjusted for LGE%, GLS, and LVEF on the basis of Model 1, the independent predictive value of CTI persisted (OR = 1.33, 95% CI: 1.13–1.56, P = 0.001). In Model 2, LGE% (OR = 1.01, 95% CI: 1.01–1.02, P < 0.001), GLS (OR = 0.95, 95% CI: 0.92–0.99, P = 0.006), hsTnT (OR = 1.51, P < 0.001), STEMI (OR = 1.53, P = 0.006), and IRA-LAD (OR = 1.67, P < 0.001) were also independent predictors of MVO.

**Table 4:** Multivariable logistic regression analysis for MVO in all patients.

Variables	Model 1		Model 2	
	OR (95%CI)	*P*	OR (95%CI)	*P*
LGE%	-	-	1.01 (1.01 ~ 1.02)	<.001
GLS, %	-	-	0.95 (0.92 ~ 0.99)	0.006
hsTnT (ng/ml)	1.52 (1.36 ~ 1.71)	<.001	1.51 (1.34 ~ 1.69)	<.001
CTI	1.48 (1.27 ~ 1.73)	<.001	1.33 (1.13 ~ 1.56)	0.001
STEMI, n (%)	1.49 (1.11 ~ 2.01)	0.008	1.53 (1.13 ~ 2.07)	0.006
IRA-LAD, n (%)	1.72 (1.31 ~ 2.26)	<.001	1.67 (1.26 ~ 2.20)	<.001

LVEF, left ventricular ejection fraction; GLS, global longitudinal strain; LGE, late gadolinium enhanced; IRA, infarct-related artery; LAD, left anterior descending artery; hs-TnT, high sensitivity troponin T; STEMI, ST-segment elevation myocardial infarction; CTI, C-reactive protein–triglycerideglucose index.

Model 1 adjusted for age, STEMI, IRA-LAD, preprocedural TIMI flow grade, diabetes mellitus, systolic blood pressure, heart rate, NT-proBNP, hsTnT, and CTI. Model 2 included all variables in Model 1, with further adjustment for LGE, GLS, and LVEF.

### Logistic regression analysis for MVO in the diabetic and non-diabetic subgroups

In the diabetic subgroup (**Table 5**), CTI was significantly associated with MVO (OR = 1.34, 95% CI: 1.03–1.74, P = 0.030). Other significant factors included age, LGE%, LVEF, GLS, NT-proBNP, and hsTnT. Notably, in the diabetic subgroup, neither the TyG index (OR = 1.11, P = 0.556) nor FBG showed a statistically significant association with MVO. In the non-diabetic subgroup, the association between CTI and MVO was more pronounced (OR = 1.65, 95% CI: 1.37–2.00, P < 0.001). In the tertile-based analysis, compared with the low-CTI group, both the middle-CTI group (OR = 1.73, P = 0.002) and the high-CTI group (OR = 2.60, P < 0.001) had significantly elevated MVO risk.

**Table 5:** Univariate logistic regression analysis for MVO.

Variables	Diabetes		Non-diabetes	
	OR (95%CI)	*P*	OR (95%CI)	*P*
Male, n (%)	1.28 (0.68 ~ 2.40)	0.447	0.97 (0.66 ~ 1.44)	0.882
Age (years)	1.03 (1.01 ~ 1.05)	0.017	1.00 (0.99 ~ 1.01)	0.765
BMI (kg/m^2^)	1.04 (0.97 ~ 1.13)	0.263	0.99 (0.95 ~ 1.03)	0.593
Smoking, n (%)	1.49 (0.90 ~ 2.45)	0.120	1.27 (0.94 ~ 1.70)	0.118
Hypertension, n (%)	0.91 (0.55 ~ 1.50)	0.713	0.92 (0.69 ~ 1.24)	0.600
Stroke, n (%)	0.73 (0.36 ~ 1.46)	0.374	0.97 (0.59 ~ 1.62)	0.916
Heart rate, bpm	1.01 (0.99 ~ 1.03)	0.188	1.02 (1.01 ~ 1.03)	0.008
SBP (mmHg)	1.00 (0.99 ~ 1.01)	0.934	0.99 (0.98 ~ 0.99)	0.011
DBP (mmHg)	1.02 (1.00 ~ 1.04)	0.055	1.00 (0.99 ~ 1.01)	0.646
LGE%	1.01 (1.01 ~ 1.02)	0.006	1.02 (1.01 ~ 1.02)	<.001
LVESVI, mL/m^2^	0.99 (0.98 ~ 1.01)	0.416	1.00 (0.99 ~ 1.01)	0.599
LVEDVI, mL/m^2^	1.01 (1.00 ~ 1.02)	0.192	1.00 (1.00 ~ 1.01)	0.550
LVEF, %	0.97 (0.94 ~ 0.99)	0.041	0.99 (0.97 ~ 1.00)	0.129
LV mass, g	1.00 (0.99 ~ 1.00)	0.274	1.00 (0.99 ~ 1.00)	0.384
GLS, %	0.93 (0.88 ~ 0.99)	0.018	0.94 (0.91 ~ 0.97)	<.001
NT-proBNP (pg/ml)	1.43 (1.14 ~ 1.79)	0.002	1.13 (0.99 ~ 1.30)	0.072
hsTnT (ng/ml)	1.65 (1.33 ~ 2.03)	<.001	1.63 (1.44 ~ 1.85)	<.001
hs-CRP, mg/L	1.01 (1.01 ~ 1.01)	0.049	1.01 (1.01 ~ 1.01)	<.001
eGFR, mL/min/1.73m^2^	0.99 (0.97 ~ 1.00)	0.158	1.00 (0.99 ~ 1.01)	0.541
CTI	1.34 (1.03 ~ 1.74)	0.030	1.65 (1.37 ~ 2.00)	<.001
TyG	1.11 (0.79 ~ 1.55)	0.556	1.32 (1.03 ~ 1.70)	0.029
FBG, mmol/L	1.03 (0.96 ~ 1.12)	0.399	1.14 (1.04 ~ 1.24)	0.006
TC (mmol/L)	1.07 (0.84 ~ 1.37)	0.576	1.06 (0.93 ~ 1.22)	0.384
TG (mmol/L)	1.07 (0.92 ~ 1.25)	0.378	1.18 (1.02 ~ 1.36)	0.027
LDL-C (mmol/L)	0.97 (0.74 ~ 1.28)	0.847	1.07 (0.91 ~ 1.26)	0.401
HDL-C (mmol/L)	1.25 (0.45 ~ 3.42)	0.669	1.50 (0.83 ~ 2.70)	0.176
Preprocedural TIMI ≤1, n (%)	1.66 (0.91 ~ 3.04)	0.099	1.84 (1.29 ~ 2.63)	<.001
CTI groups, n (%)				
Low CTI	Reference		Reference	
Medium CTI	0.88 (0.39 ~ 1.98)	0.753	1.73 (1.22 ~ 2.46)	0.002
High CTI	1.38 (0.67 ~ 2.85)	0.380	2.60 (1.78 ~ 3.80)	<.001
STEMI, n (%)	1.54 (0.90 ~ 2.64)	0.117	2.20 (1.60 ~ 3.03)	<.001
MACE, n (%)	2.65 (1.47 ~ 4.77)	0.001	2.13 (1.51 ~ 3.00)	<.001
IRA-LCX, n (%)	1.10 (0.57 ~ 2.13)	0.769	0.65 (0.44 ~ 0.95)	0.026
IRA-LAD, n (%)	1.46 (0.89 ~ 2.41)	0.137	0.65 (0.44 ~ 0.95)	0.026
IRA-RCA, n (%)	0.69 (0.41 ~ 1.18)	0.173	0.79 (0.57 ~ 1.09)	0.143
IRA-LM, n (%)	0.00 (0.00 ~ Inf)	0.981	0.00 (0.00 ~ Inf)	0.972
IRA-RM, n (%)	0.00 (0.00 ~ Inf)	0.987	0.00 (0.00 ~ Inf)	0.972
Killip class>1, n (%)	1.40 (0.57 ~ 3.42)	0.466	1.59 (0.93 ~ 2.71)	0.087
Statins, n (%)	0.58 (0.15 ~ 2.30)	0.439	0.85 (0.45 ~ 1.58)	0.598
SGLT2 inhibitor, n (%)	0.93 (0.55 ~ 1.56)	0.787	2.30 (1.47 ~ 3.61)	<.001
β-blockers, n (%)	0.79 (0.35 ~ 1.81)	0.584	1.25 (0.83 ~ 1.88)	0.288
ACEI/ARB, n (%)	0.79 (0.47 ~ 1.33)	0.374	0.84 (0.62 ~ 1.13)	0.251
Aspirin, n (%)	0.87 (0.33 ~ 2.33)	0.784	0.94 (0.55 ~ 1.62)	0.837
P2Y12, n (%)	0.58 (0.15 ~ 2.30)	0.439	0.89 (0.47 ~ 1.67)	0.720

BMI, body mass index; GFR, glomerular filtration rate; LVEF, left ventricular ejection fraction; GLS, global longitudinal strain; LV, left ventricular; EDVi, end-diastolic volume index; LGE, late gadolinium enhanced; ESVi, end-systolic volume index; SBP, systolic blood pressure; DBP, diastolic blood pressure; IRA, infarct-related artery; LAD, left anterior descending artery; LCX, left circumﬂex artery; RCA, right coronary artery; LM, left main coronary artery; RM, right main coronary artery; HDL-C, high-density lipoprotein cholesterol; LDL-C, low-density lipoprotein cholesterol; hs-CRP, high sensitivity C-reactive protein; hs-TnT, high sensitivity troponin T; NT-proBNP, N-terminal pro-B-type natriuretic peptide; ACEI, angiotensin-converting-enzyme inhibitor; ARB, angiotensin II receptor; SGLT2, sodium-dependent glucose transporters 2; TC, total cholesterol; TG, triglycerides; STEMI, ST-segment elevation myocardial infarction; TyG, triglyceride-glucose index; TIMI, Thrombolysis in Myocardial Infarction flow grade; MACE, major adverse cardiovascular events; FBG, fasting blood glucose; CTI, C-reactive protein–triglyceride glucose index.

In the multivariable logistic regression in the diabetic subgroup, IS (OR = 1.01, 95% CI: 1.00–1.02, P = 0.015), GLS (OR = 0.94, 95% CI: 0.88–1.00, P = 0.043), hsTnT (OR = 1.60, 95% CI: 1.29–1.98, P < 0.001), and IRA-LAD (OR = 1.84, 95% CI: 1.33–2.55, P < 0.001) were independent factors of MVO. In the non-diabetic subgroup, CTI remained an independent risk factor for MVO (OR = 1.38, 95% CI: 1.12–1.71, P = 0.003). LGE% (OR = 1.01, P < 0.001), GLS (OR = 0.96, P = 0.030), hsTnT (OR = 1.49, P < 0.001), STEMI (OR = 1.77, P = 0.002), and IRA-LAD (OR = 1.84, P < 0.001) were also independent factors (**Table 6**).

**Table 6:** Multivariable logistic regression analysis for MVO.

Variables	Diabetes		Non-diabetes	
	OR (95%CI)	*P*	OR (95%CI)	*P*
LGE%	1.01 (1.00 ~ 1.02)	0.015	1.01 (1.01 ~ 1.02)	<.001
GLS, %	0.94 (0.88 ~ 1.00)	0.043	0.96 (0.92 ~ 1.00)	0.030
hsTnT (ng/ml)	1.60 (1.29 ~ 1.98)	<.001	1.49 (1.30 ~ 1.71)	<.001
CTI	-	-	1.38 (1.12 ~ 1.71)	0.003
STEMI, n (%)	-	-	1.77 (1.24 ~ 2.53)	0.002
IRA-LAD, n (%)			1.84 (1.33 ~ 2.55)	<.001

LVEF, left ventricular ejection fraction; GLS, global longitudinal strain; LGE, late gadolinium enhanced; IRA, infarct-related artery; LAD, left anterior descending artery; hs-TnT, high sensitivity troponin T; STEMI, ST-segment elevation myocardial infarction; CTI, C-reactive protein–triglycerideglucose index.

In patients with diabetes, adjustments were made for age, STEMI, IRA-LAD, pre-procedural TIMI flow grade, LGE, GLS, LVEF, NT-proBNP, hsTnT, and CTI. In patients without diabetes, adjustments were made for age, STEMI, IRA-LAD, pre-procedural TIMI flow grade, LGE, GLS, LVEF, NT-proBNP, hsTnT, CTI, systolic blood pressure, and heart rate.

### Dose-response relationship between CTI and MVO

**Figure 2:**
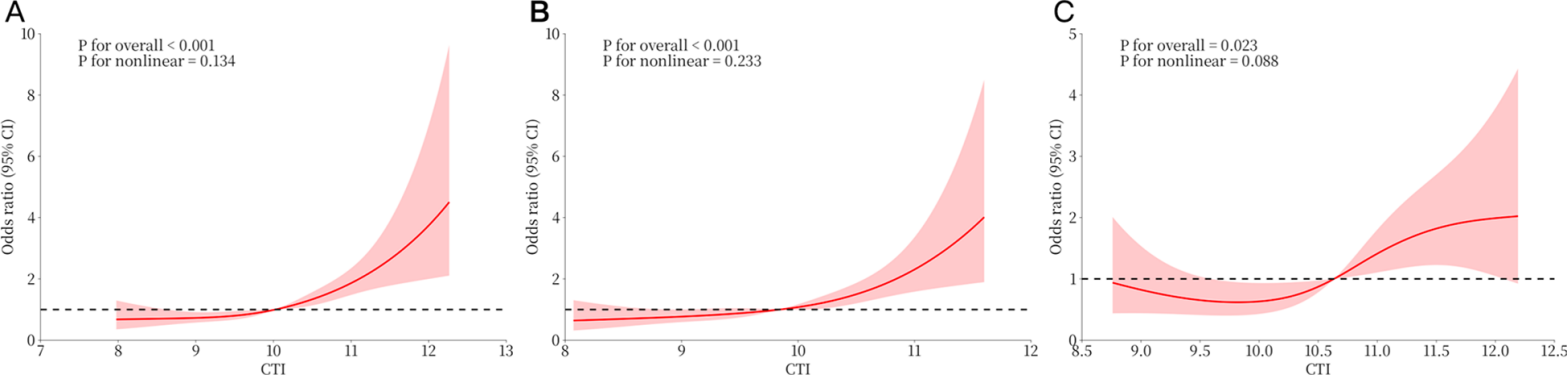
Dose-response relationship between CTI and MVO. **(A)** Dose-response relationship between CTI and MVO in all patients. **(B)** Dose-response relationship between CTI and MVO in patients without diabetes. **(C)** Dose-response relationship between CTI and MVO in patients with diabetes. MVO, microvascular obstruction; CTI, C-reactive protein-triglyceride glucose index.

RCS analysis demonstrated a significant dose-response relationship between CTI and MVO risk in the overall population (P for overall < 0.001), with a linear trend (P for nonlinear = 0.134), indicating that MVO risk increased proportionally with each increment in CTI. In both the diabetic subgroup (P for overall < 0.001, P for nonlinear = 0.233) and the non-diabetic subgroup (P for overall = 0.023, P for nonlinear = 0.088), significant linear dose-response relationships between CTI and MVO were also observed (**Figure 2**).

### Internal validation of the MVO identification model

Internal validation of the MVO identification model was performed using 200 bootstrap resamples. The apparent AUC of the model was 0.736, and the optimism-corrected AUC was 0.722, indicating acceptable internally validated discriminative performance. The bootstrap-corrected calibration intercept was -0.001, and the calibration slope was 0.925, suggesting generally acceptable agreement between predicted and observed MVO risk.

### Kaplan-Meier analysis of CTI and long-term MACE

**Figure 3:**
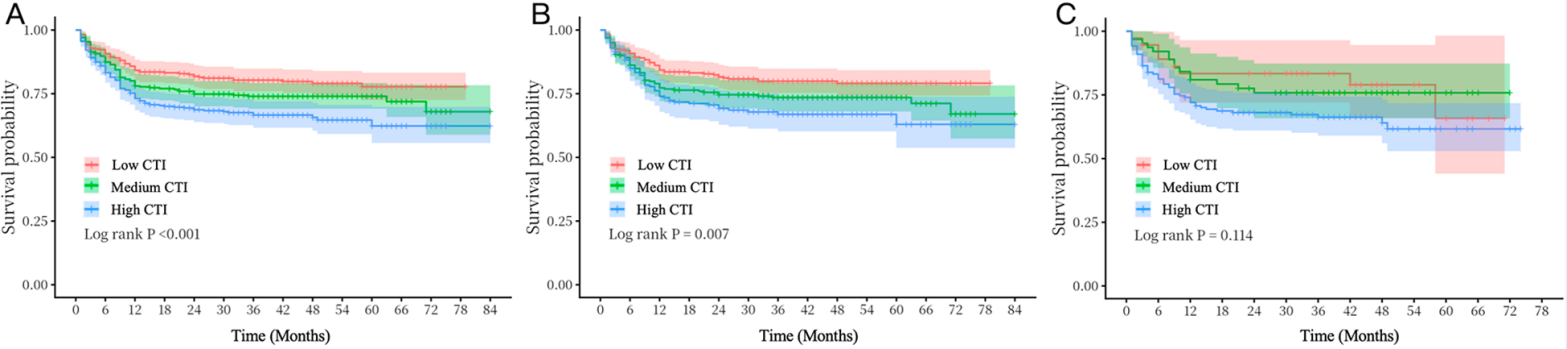
Kaplan-Meier curve for patients based on the CTI. **(A)** Kaplan-Meier curve of CTI for MACE in all patients. **(B)** Kaplan-Meier curve of CTI for MACE in patients without diabetes. **(C)** Kaplan-Meier curve of CTI for MACE in patients with diabetes.

During a median follow-up of 43 (33, 56) months, a total of 256 (26.5%) patients experienced MACE (**Supplementary Table 1**). Kaplan-Meier survival analysis showed that in the overall population, MACE-free survival rates differed significantly among the CTI groups (log-rank P = 0.001), with the highest long-term MACE risk in the high-CTI group (MACE incidence 33.04%) and the lowest in the low-CTI group (19.88%). In the non-diabetic subgroup, higher CTI levels were also significantly associated with worse MACE-free survival (log-rank P = 0.007). In the diabetic subgroup, however, the difference in MACE-free survival among the three groups did not reach statistical significance (log-rank P = 0.114) (**Figure 3**).

### Cox regression and time-dependent ROC analysis for long-term MACE

In Cox proportional hazards regression analysis, CTI as a continuous variable was significantly associated with long-term MACE in the overall population (HR = 1.368, 95% CI: 1.204–1.554, P < 0.001). After adjustment for age, sex, STEMI, diabetes mellitus, systolic blood pressure, heart rate, NT-proBNP, and hsTnT, CTI remained independently associated with MACE (HR = 1.296, 95% CI: 1.123–1.495, P < 0.001). In the non-diabetic subgroup, CTI also remained independently associated with MACE after multivariable adjustment (HR = 1.367, 95% CI: 1.137–1.642, P < 0.001). However, in the diabetic subgroup, the association between CTI and MACE was attenuated and no longer statistically significant after adjustment (HR = 1.119, 95% CI: 0.885–1.416, P = 0.348) (**Supplementary Table 2**). Time-dependent ROC analysis showed that the AUC values of CTI for predicting MACE at 1, 2, and 3 years were 0.591, 0.604, and 0.621 in the overall population, respectively. In the non-diabetic subgroup, the corresponding AUC values were 0.588, 0.602, and 0.620, respectively. In the diabetic subgroup, the AUC values were 0.595, 0.601, and 0.615, respectively. These findings suggest that CTI has modest prognostic discrimination for long-term MACE, particularly in the overall and non-diabetic populations (**Supplementary Table 2**).

## Discussion

This study adds to the limited evidence regarding the association between CTI and MVO following PCI in AMI patients. Our principal findings include: (1) CTI is an independent risk factor for MVO, with significant associations maintained in the overall population and in both diabetic and non-diabetic subgroups; (2) RCS analysis revealed a significant linear dose-response relationship between CTI and MVO risk, consistent across the overall population and both subgroups; and (3) in the overall population and non-diabetic patients, higher CTI levels were significantly associated with increased long-term MACE risk, whereas no statistically significant difference was observed in the diabetic subgroup.

The TyG index, a surrogate marker of insulin resistance, has been linked to coronary microvascular dysfunction and cardiovascular events, while CRP is a well-established marker of systemic inflammation and cardiovascular risk (20, 21). Compared with either marker alone, CTI integrates metabolic and inflammatory information into a single composite indicator. Previous studies have shown that CTI is closely associated with adverse cardiovascular outcomes (11–14). Our findings extend previous evidence by showing that CTI is associated with CMR-defined MVO, a direct imaging marker of myocardial microvascular injury, in AMI patients after PCI. The association between CTI and MVO can be explained through multiple pathophysiological mechanisms. First, IR can suppress multiple signaling pathways, reduce the activity of endothelial nitric oxide synthase and the production of nitric oxide, thereby impairing microvascular vasodilation and leading to endothelial dysfunction (22). Furthermore, hyperinsulinemia associated with IR elevates free fatty acid and reactive oxygen species (ROS) levels, disrupts lipid metabolism, and consequently increases oxidative stress and promotes the release of pro-inflammatory cytokines, all of which contribute to microvascular dysfunction (23). Second, CRP increases the expression of adhesion molecules on endothelial cells, promotes leukocyte adhesion and migration, and enhances local inflammation through activation of the classical complement pathway, thereby contributing to plaque instability and accelerating thrombus formation (24, 25). More importantly, clinical and epidemiological evidence indicates that IR and inflammation exert synergistic effects at the molecular and cellular levels, mutually reinforcing each other (26). In addition, RCS analysis further revealed a significant linear dose-response relationship between CTI and MVO risk. This linear dose-response pattern suggests a stable and predictable association between CTI and MVO, whereby MVO risk increases proportionally with each increment in CTI, without a significant threshold effect or nonlinear inflection point. This finding is consistent with previous studies reporting linear relationships between CTI and cardiovascular disease mortality and stroke risk (27, 28).

An important finding of this study is the differential predictive effect of CTI on MVO and MACE between the diabetic and non-diabetic subgroups. CTI showed a more consistent independent association with MVO and adverse MACE-free survival in non-diabetic patients, whereas these associations were attenuated in diabetic patients. This discrepancy may be related to several factors. First, diabetes itself is a potent metabolic disorder involving multiple complex mechanisms that may increase MVO risk through pathways independent of IR and CRP, thereby attenuating the incremental predictive value of CTI (13, 29). Second, there is substantial variability in glycemic control among diabetic patients; the pathological features of poorly controlled diabetes may differ significantly from those of well-controlled diabetes, and the severity of chronic hyperglycemia is directly related to myocardial microvascular injury, which may partially mask the correlation between CTI levels and MVO risk (30, 31). Additionally, the relatively limited sample size of the diabetic subgroup in this study may have reduced the statistical power to detect the independent effect of CTI. Finally, given the strong predictive value of MVO for MACE risk, longer follow-up periods may yield different results.

In response to this prognostic heterogeneity, the additional Cox regression analysis confirmed that CTI was independently associated with long-term MACE in the overall and non-diabetic populations. However, the time-dependent ROC analysis showed only modest discriminative performance, suggesting that CTI should be interpreted as a clinically accessible risk-associated biomarker rather than a standalone prognostic model. Given that CTI is derived from routine laboratory parameters, it may serve as a clinically accessible risk-associated biomarker for identifying patients at higher risk of MVO and adverse long-term outcomes after AMI. However, CTI should not be interpreted as a standalone prognostic model or a direct basis for clinical decision-making. Its potential utility should be further evaluated in prospective studies and externally validated cohorts.

## Limitations

Several limitations of this study should be acknowledged. First, this was a single-center retrospective cohort analysis; despite extensive covariate adjustment in the statistical analyses, unmeasured residual confounders may still have influenced the results. Moreover, in clinical practice, patients with severe heart failure or limited ability to cooperate may be unable to tolerate or complete CMR examination, which may have led to the exclusion of patients with more severe clinical conditions and poorer prognosis, thereby limiting the generalizability of our findings to the broader AMI population. Second, CTI was calculated based on laboratory measurements at a single time point and cannot reflect the longitudinal association between dynamic CTI changes and MVO risk. Future multicenter, large-scale prospective longitudinal cohort studies with repeated measurements during follow-up are needed to further validate the clinical impact of dynamic CTI on cardiovascular outcomes. Third, although our study investigated the relationship between CTI levels and MVO as well as MACE in AMI patients, the specific mechanisms underlying IR and inflammation require further elucidation through basic research. Finally, although bootstrap internal validation was performed and showed acceptable internally validated performance of the MVO identification model, the present study lacked external validation. Therefore, the generalizability and transportability of the model require further confirmation in independent multicenter cohorts.

## Conclusion

CTI is significantly and independently associated with MVO following PCI in AMI patients, with a stable linear dose-response relationship. In the overall population and non-diabetic patients, higher CTI levels are significantly associated with increased long-term MACE risk. These findings suggest that CTI may serve as a clinically accessible risk-associated biomarker for post-AMI risk stratification, pending external validation in independent prospective cohorts.

## Data Availability

The raw data supporting the conclusions of this article will be made available by the authors, without undue reservation.
