# Supplementary material for: Association of the C-reactive protein-triglyceride glucose index with microvascular obstruction and long-term prognosis in patients with acute myocardial infarction: a CMR-based study
Source: Front Endocrinol (Lausanne). 2026 Jul 15;17:1889147. doi: 10.3389/fendo.2026.1889147 (PMC13414900; doi:10.3389/fendo.2026.1889147)
Supplement: Supplementary file 1 [file Table1.docx]

**Table S1** Adverse events during follow-up

| Adverse events, n (%) | AMI (967) |
| --- | --- |
| MACE | 256 (26.5) |
| All-cause death | 33 (3.4) |
| Recurrent myocardial infarction | 46 (5.8) |
| Stroke | 42 (4.3) |
| Hospitalization for heart failure | 135 (14.0) |

AMI = acute myocardial infarction, MACE = major adverse cardiac events.
