# Supplementary material for: Association of the C-reactive protein-triglyceride glucose index with microvascular obstruction and long-term prognosis in patients with acute myocardial infarction: a CMR-based study
Source: Front Endocrinol (Lausanne). 2026 Jul 15;17:1889147. doi: 10.3389/fendo.2026.1889147 (PMC13414900; doi:10.3389/fendo.2026.1889147)
Supplement: Supplementary file 2 [file Table2.docx]

**Table S2** Cox regression and time-dependent ROC analysis of CTI for MACE

| Population | Cox model | HR | 95% CI | P value | 1-year AUC | 2-year AUC | 3-year AUC |
| --- | --- | --- | --- | --- | --- | --- | --- |
| Overall | Univariable | 1.368 | 1.204 ~ 1.554 | <0.001 | 0.591 | 0.604 | 0.621 |
| Overall | Multivariable | 1.296 | 1.123 ~ 1.495 | <0.001 | - | - | - |
| Non-diabetic | Univariable | 1.420 | 1.193 ~ 1.690 | <0.001 | 0.588 | 0.602 | 0.620 |
| Non-diabetic | Multivariable | 1.367 | 1.137 ~ 1.642 | <0.001 | - | - | - |
| Diabetic | Univariable | 1.304 | 1.042 ~ 1.632 | 0.021 | 0.595 | 0.601 | 0.615 |
| Diabetic | Multivariable | 1.119 | 0.885 ~ 1.416 | 0.348 | - | - | - |

AUC = area under the curve; CI = confidence interval; CTI = C-reactive protein–triglyceride glucose index; HR = hazard ratio; MACE = major adverse cardiovascular events; ROC = receiver operating characteristic; STEMI = ST-segment elevation myocardial infarction.
